# Author's reply regarding “Subtalar extra-articular screw arthroereisis: Early North American experience in a novel minimally invasive treatment for pediatric pes planovalgus”

**DOI:** 10.1016/j.jposna.2024.100034

**Published:** 2024-03-29

**Authors:** Susan T. Mahan

**Affiliations:** Harvard Medical School, Department of Orthopaedics, Boston Children’s Hospital, Boston, MA, USA

Dear Editors,

Thank you very much for sharing the letter by Julio de Pablos outlining the history of the “calcaneo-stop” (also known as Subtalar Extra-articular Screw Arthroereisis or SESA) procedure. This is most enlightening! While I had tried to research the history, I was unable to discover the work of Dr. Recaredo Alvarez Rodriguez. I appreciate this history lesson and will forever be grateful for the work of Dr. Alvarez Rodriguez to pioneer this technique. Hopefully, with your letter included in *JPOSNA®* and the English literature, his work will be recognized. With this ongoing discussion, we hope to continue to expand this procedure to North America and beyond so many children with painful flatfeet can benefit from his insights.

Thank you very much for sharing!

## Declaration of conflicts of interest

The authors have no conflicts of interest to disclose.

